# Supplementary material for: Are carbon emissions trading and green financial instruments synergistic? -Comprehensive quantitative research based on content analysis
Source: PLoS One. 2024 Mar 7;19(3):e0298601. doi: 10.1371/journal.pone.0298601 (PMC10919601; doi:10.1371/journal.pone.0298601)
Supplement: S1 Data — (ZIP) [file pone.0298601.s001.zip › Data/地级市Z_F工作报告.zip!/╜¡╦╒/╤╬│╟╩╨/└┤╫╘╣1⁄2╓┌║┼í╛┬φ┐╦╩2╛▌═°í┐_2019.docx]

各位代表：

现在，我代表盐城市人民政府向大会作工作报告，请予审议，并请各位政协委员和其他列席人员提出意见。

2018年工作回顾

过去的一年，面对错综复杂的宏观形势，我们认真贯彻落实习近平新时代中国特色社会主义思想和党的十九大精神，按照中央和省市委决策部署，坚持稳中求进工作总基调，全面贯彻新发展理念，围绕“两高”目标、“三市”战略，深入践行“两海两绿”发展路径，全力打好“三大攻坚战”，统筹做好稳增长、促改革、调结构、惠民生、防风险、保稳定各项工作，经济社会发展稳中有进，高质量发展开局良好，较好地完成了市八届人大二次会议确定的目标任务。全市地区生产总值增长5.5%，固定资产投资增长9.4%，一般公共预算收入381亿元、增长5.8%，其中税收收入305亿元、增长12.2%，税收占比80.1%。注册外资实际到账9.13亿美元，增长15.8%；外贸进出口总额95.5亿美元，增长10.4%。全口径工业开票销售5528亿元，增长17%。城乡居民人均可支配收入分别达到35831元、20358元，增长8.2%和8.8%。居民消费价格指数涨幅1.9%。

现代产业体系加快建设。持续开展“产业项目载体提升年”活动，“两重一实”项目完成投资1100亿元，新开工亿元以上产业项目270个、竣工185个，制造业投资占固定资产投资比重63%。主动承接长江经济带产业转移，宝武集团2000万吨级绿色精品钢生产基地落户盐城，实现了重特大产业项目的历史性突破。东山精密、中天光纤、阿特斯光伏电池、富乐德半导体等重大项目竣工投产。提档升级传统产业，510个千万元以上技改项目完成投资428亿元，新增省级智能车间示范项目9个，300家规模以上企业“上云”。东风悦达起亚焕驰轿车出口埃及、菲律宾，迈出开辟海外汽车市场第一步。培育战略性新兴产业，全年产值增长22%，占工业总产值比重23.7%。新能源发电量占全市用电量比重39%，海上风电总装机容量150万千瓦，居全国首位。加快发展现代服务业，服务业增加值增长8.1%，对经济发展贡献率50%以上，社会消费品零售总额增长6.8%。新增省级生产性服务业集聚示范区3家，创成国家4A级旅游景区4家。全社会存款6421亿元，增长3.3%；贷款余额4998亿元，增长16.6%。

创新发展能力显著增强。强化企业创新主体作用，新增高新技术企业285家，金风科技建成风电行业国家地方联合工程实验室，新创国家级企业技术中心2家、国家知识产权优势企业54家，新增省级企业研发机构44家、“专精特新”产品27个、“科技小巨人”企业13户，签约和实施产学研合作项目1140项。全社会研发投入占GDP比重2.1%，提高0.07个百分点。高新技术产业产值占规模以上工业产值比重37%。万人发明专利拥有量7.59件。围绕创新链做实资金链，设立产业投资基金12支，总规模260亿元。持续实施“515”人才引进计划，出台人才激励“10条政策”，发放人才资助资金1.12亿元，引进各类领军人才361名。

沿海发展水平不断提升。深入推进沿海开发，盐城港新增万吨级以上码头泊位3个，全年吞吐量9500万吨。滨海港区30万吨级深水航道等工程列入淮河生态经济带发展规划。获批国家海洋经济发展示范区。以接轨上海推动开放沿海，组织1026家单位参加首届中国国际进口博览会，在上海成功举办系列专题推介活动，盐城（上海）国际科创中心入驻科技型企业16家。实施新一轮基础设施建设三年行动计划，完成年度投资638亿元。盐青铁路正式通车，盐城驶入“高铁时代”。盐徐高铁完成线下工程，盐通高铁桥梁桩基工程任务过半。南洋国际机场T2航站楼建成运营，新增和恢复香港、长春等航线，全年旅客吞吐量182万人次。盐城至南京等5条高速公路列入省高速公路网规划。水利、电力、通信等基础设施建设得到新的加强。

乡村振兴战略全面实施。推动农村产业兴旺，调减低效粮田74.2万亩，粮食总产量140.7亿斤，新增设施农（渔）业14.5万亩，兴办投资500万元以上农业龙头企业60家，培育销售超千万元农业电商企业53家，获批省现代农业科技园区4家，绿色优质农产品比重38%，响水荣获“中国西兰花之乡”称号，东台、大丰通过全国农产品质量安全县验收。促进城乡深度融合，常住人口城镇化率64.03%。开展农村环境整治提升行动，建成省美丽乡村20个，大丰新丰镇全心村获评“中国美丽休闲乡村”。新改建农村公路1418公里、桥梁801座，15个镇新开镇村公交，行政村双车道四级公路实现全覆盖。完成农村康居工程三年行动计划，累计改造危旧房18万户，开展农民群众住房条件改善工作并启动“十镇百村”试点。深化农村土地“三权分置”、农村产权制度改革，新建家庭农场563家，新增农民专业合作社493家。

改革开放力度持续加大。深化供给侧结构性改革，帮助企业降低成本38.9亿元，实现直接融资398.5亿元，新增新三板企业3家，丰山集团在A股成功上市。推进“放管服”改革，“3550”改革实现常态化、制度化，在全国首家全面推行“三书合一”，在全省首推投资建设项目不见面图审改革。调整优化国有资产布局，完善国有企业运行监管体制。加强政府性债务管理，严格控制新增债务，妥善化解存量债务。税收征管体制改革顺利推进。中韩（盐城）产业园建设实施方案获省政府批准，中韩（盐城）产业园发展基金引导投资76.3亿元，其中撬动社会资本59.6亿元。中韩产业园合作协调机制第二次会议、第一届中韩产业园合作交流会和第六届中韩公共外交论坛在盐城召开。推动园区等级创建，滨海港工业园区加快建设，射阳港经济开发区成为省级开发区，建湖高新区、盐南高新区、盐城环保高新区获批省级高新区。

中心城市功能更加完善。突出规划引领，修编新一轮城市总体规划，实施城建重点项目189项，完成投资354亿元。推进“五大组团”建设，南海未来城组团开工建设南海公园等项目，大洋湾生态组团建成登瀛阁、长乐水世界等项目，高铁枢纽组团启动建设高铁站房，先锋岛生态组团完成部分地块改造建设，斗龙港生态组团建成城市森林公园一期项目。开展“三治三化”专项行动，全市改造棚户区3.36万户、475.4万平方米，其中市区改造棚户区1.87万户、251万平方米，整治6个老旧小区、30万平方米，拆除各类违法建设54.2万平方米。新四军重建军部纪念塔（大铜马）复位，启动建军路商圈改造。市区内环高架三期主体工程完成50%以上。开工建设盐渎路跨通榆河大桥，打通一批“断头路”。按照“十没有”“二十有”标准实施城市精细化管理，开展工地围挡、户外广告、停车秩序整治行动，全国文明城市创建深入推进。

生态环境治理扎实有效。坚决打好污染防治攻坚战，贯彻中央环保督察整改要求，推进“263”“绿盾2018”等专项行动，抓好十大挂牌督办工程，实行环境保护行政执法与司法联动，依法有力处置辉丰公司污染违法等问题，一批历史遗留问题得到有效解决。新关停化工生产企业22户、危化品码头4个，化工生产企业入园率85%，保持全省第一。大力推进节能减排，非电行业煤炭消费比2016年减少97万吨，创建国家绿色工厂3家、国家绿色园区1家。PM2.5年均浓度43微克/立方米。整治城市黑臭河道21条，城镇污水集中处理率84%，地表水国省考断面消灭劣Ⅴ类。全面推行河长制，实施21条河湖“一河一策”整治行动，全国水生态文明城市试点建设通过验收。大型规模畜禽养殖场治理率100%。推广垃圾分类处理，加强固体废弃物、有毒化学品和垃圾处置，危废处置能力新增6.2万吨。国务院正式同意我市黄海湿地项目申报世界自然遗产，接受世界自然保护联盟现场考察评估。成功举办2018黄（渤）海湿地盐城国际会议。创建国家森林城市，新增成片林15.45万亩、改造既有林16.8万亩，建成森林小镇24个、森林村庄112个，城镇绿地新增3447公顷，绿化造林总量全省第一。建湖创成国家生态文明建设示范县，九龙口景区获批国家湿地公园。

民生幸福指数稳步提高。坚持民生优先发展，民生类支出占财政支出比重77%，20件民生实事完成年度投资173.7亿元。新水源地及跨区域引水工程建成通水，在全省率先完成全市域水源地达标整治，“一桶水”工程全面完成。打好精准脱贫攻坚战，14.5万建档立卡低收入人口和37个省定经济薄弱村实现脱贫，农村贫困人口年人均纯收入全部达到国家4000元的脱贫标准，消除绝对贫困现象。促进就业创业，新增城镇就业11万人，城镇登记失业率控制在1.8%。加快教育事业发展，盐城工学院获批硕士学位授予单位，新建、改扩建各类学校66所，重奖名师、引进名校，普通高中录取率65.3%，提高10个百分点，教育领域突出问题专项治理取得明显成效。推动“健康盐城”建设，新设立院士工作站2个、名院名医工作室8个，新增省级临床重点专科7个，新创省示范镇卫生院11个、示范村卫生室133个。实施文化惠民工程，全面完成村（社区）综合文化服务中心建设任务，淮剧《送你过江》荣获省紫金文化艺术节优秀剧目奖。新当选“中国好人”11名。阜宁成为全国新时代文明实践中心试点县。市区新建健身步道25公里，改造50个老旧小区体育设施。参加第19届省运动会，成功举办中华龙舟赛等赛事活动。健全社会保障体系，实施全市统一的城乡居民医疗保险制度，提高城乡居民低保标准，在苏北率先实现城乡一体化。深化法治盐城、平安盐城建设，大力开展扫黑除恶专项斗争，组织系列“黄海行动”和“梳网清格行动”，连续7年获评省社会治安综合治理工作先进市。扎实抓好信访稳定，进京、去省非访数持续下降。强化安全生产监督管理，事故起数和死亡人数继续保持双下降。民族团结进步活动广泛开展，宗教工作进一步加强。支持驻盐部队建设，落实拥军优属政策，军政军民团结更加巩固。

一年来，我们加强政府自身建设，自觉接受市人大依法监督、市政协民主监督，认真听取民主党派、工商联、人民团体和各界人士意见，办理市人大代表议案建议162件、市政协委员提案346件，办结率100%，满意和基本满意率99.8%。审计、统计、物价、供销社、公积金、人民防空、防灾减灾、食品药品、质监等工作得到加强，外事、侨务、对台、档案、科协、地方志、妇女、儿童、青少年、老龄、慈善、残疾人、红十字等工作取得新的进展。

各位代表，过去一年的成绩来之不易，这是省委、省政府和市委正确领导的结果，是全市人民不断解放思想、改革创新，锐意进取、团结奋斗的结果。在此，我谨代表市人民政府，向广大干部群众，向全体人大代表、政协委员，向离退休老同志，向各民主党派、工商联、人民团体和各界人士，向驻盐人民解放军、武警官兵、公安干警和省属各单位，向所有关心和支持盐城发展的海内外朋友，致以崇高的敬意和衷心的感谢！

在肯定成绩的同时，我们也清醒地看到，当前经济社会发展还存在一些问题：经济结构还不够优化，发展活力与动力还不足，经济密度不高，科技创新支撑不够；中心城区服务能力还不强，市县经济联系不够紧密，实现乡村振兴任重道远；民生领域还存在短板，公共服务保障不均衡，环境保护压力依然较大；政府服务效能还不高，自身能力水平还有不少差距，营商环境有待进一步优化，等等。对这些问题，我们一定高度重视，采取有力有效措施，切实加以解决。

2019年工作安排

2019年是新中国成立70周年，是决胜全面建成小康社会的关键之年。今年政府工作的总体要求是：坚持以习近平新时代中国特色社会主义思想为指导，全面贯彻党的十九大、十九届二中、三中全会、中央经济工作会议和省市委全会部署，统筹推进“五位一体”总体布局，协调推进“四个全面”战略布局，坚持稳中求进工作总基调，坚持新发展理念，坚持推动高质量发展，坚持以供给侧结构性改革为主线，坚持深化市场化改革、扩大高水平开放，加快建设现代化经济体系，继续打好“三大攻坚战”，按照“产业强市、生态立市、富民兴市”战略，走好“两海两绿”路径，进一步稳就业、稳金融、稳外贸、稳外资、稳投资、稳预期，统筹推进稳增长、促改革、调结构、惠民生、防风险、保稳定各项工作，为高水平全面建成小康社会收官打下决定性基础，推动高质量发展走在苏北苏中前列。

今年全市经济社会发展主要预期目标是：地区生产总值增长6.5%以上，一般公共预算收入增长6.5%，固定资产投资增长8%，全社会研发投入占GDP比重2.16%，社会消费品零售总额增长8%。城乡居民收入分别增长8.2%和8.5%，居民消费价格指数涨幅控制在3%左右。节能减排完成省定目标。外贸进出口和利用外资稳中提效，营商环境指数位居苏北苏中前列。

当前，宏观形势复杂多变，需要积极应对，主动作为。我们必须始终高举新时代改革开放旗帜，认真落实省委“进、稳、新、深”工作要求，坚定改革开放再出发的信念，汇聚强大力量，站稳人民立场，持续发力、协同发力、精准发力，将改革进行到底。通过深化改革破解难题、拓展空间、促进发展，坚持向改革要动力，用改革推动“进”，以改革确保“稳”，努力实现各项工作稳中有进、稳中快进、稳中优进，确保完成全年目标任务。

一、深入践行创新发展理念，培育经济增长新动能

推动制造业高质量发展。围绕建设现代化产业体系，聚焦优势主导产业，整合产业链资源，加大招商力度，提升制造业发展水平。坚定不移推动汽车产业转型发展，积极应对传统汽车市场下滑和新能源汽车快速成长的市场变化，发展自主品牌汽车、新能源汽车、智能网联车、汽车零部件和汽车后市场，引进更多的汽车领军企业，加快向“三个千亿”目标迈进。支持东风悦达起亚开拓市场，支持悦达集团培育以汽车为主的核心竞争力。推动新能源产业打造全产业链，主攻科技研发、核心部件、运维服务等高附加值环节，推进阿特斯、天合等高效光伏电池和组件项目建设，促进光伏发电向设备制造、能源物联网发展，强化智能微电网建设和利用；支持金风科技、远景能源等项目建设，大力发展海上风电，培育科研创新能力，打造新能源创新发展示范城市。推动电子信息产业高端发展，抢抓5G和新一代信息技术发展机遇，聚焦智能终端、集成电路、人工智能等领域，积极招引行业龙头项目，加快东山精密、领胜科技等重点项目建设。推动钢铁产业加快布局，全力服务宝武精品钢、德龙不锈钢等项目建设。推动机械、纺织、化工等传统产业转型升级，落实智能制造发展计划，实施500个千万元以上技改项目，新增500家规模企业“上云”，增创新的发展优势。

积极培育现代服务业。推动先进制造业与现代服务业融合发展，实施物流业发展行动计划，建设公铁水联运的物流基地、港口物流园和空港经济区，开辟对韩货运全货机业务，提升内河航道运输能力，培育汽车、钢材、建材等市场群，构建现代物流集疏运体系。积极发展都市经济，引导行业相近、类型相同的企业集群发展，培育总部经济、数字经济、平台经济等新业态，打造一批主题楼宇、高产楼宇。推动房地产市场健康有序发展。提升金融商贸能级，发展现代金融、商贸流通、教育医疗、育幼养老、文化旅游等服务业，推进建军路商圈改造、金融城二期建设和高铁枢纽片区开发等项目，优化整合消费市场，提升城市商业魅力。发展大数据产业，加快载体平台和重大项目建设，强化大数据商业服务能力。

着力增强产业创新能力。完善科技创新体系，创建国家创新型城市。围绕优势产业突破创新能力，发挥企业主体作用，推动规模以上企业普遍设立研发机构，强化核心技术研究开发。开展知识产权强企行动，高新技术企业数突破1000家。加强产学研合作，实施30项重点研发和科技成果转化项目，推进江苏新能源汽车研究院等平台建设，培育科技孵化器、众创空间，促进科技与产业深度融合。深化企业“争星创优”活动，实施“千企升级”计划，支持企业技术改造、兼并重组、挂牌上市，培育一批“专精特新”产品和“科技小巨人”企业。落实人才激励“10条政策”，深入推进“双招双引”工程，开展星级企业家能力提升、新生代企业家培育等工作，新增省级“双创”示范基地2家。加快培育创新文化，营造更加浓厚的创新氛围。

全力推进重大项目建设。大力实施“两重一实”项目，坚持双月现场观摩推进，完善领导挂钩和协调会办机制，突出产业导向、投资规模、建设进度、产出效益等重点，开展重大项目等级评价，树立高质量推进项目的鲜明导向。围绕重点领域，推进对韩日合作，深化与欧美、港澳台经贸交流，加强与北京、上海、深圳等城市产业对接，瞄准国内外行业先进企业，强化专题招商、以商引商和产业链招商，全年新签约10亿元以上产业项目25个。

二、深入践行协调发展理念，构建全域统筹新格局

坚持规划引领。完成新一轮城市总体规划修编，推动“多规合一”，指导做好县域、县城、集镇和乡村建设规划，构建现代化城镇体系，促进市与县互动并进、城与乡深度融合，引领城乡一体化高质量发展。优化城市空间布局，建设盐丰快速通道，实施通榆河、串场河沿线景观提升工程，沿新长铁路向南连接大丰推进盐丰一体化，向北延伸至新兴、上冈建设物流集聚区拓展城市功能，打造城市南北向产业发展轴线；向西建设高水平的科创园区，向东联动空港经济区、环保科技城，沿蟒蛇河、新洋港串联珍禽保护区、大洋湾景区、先锋岛生态组团和大纵湖旅游景区，构筑城市东西向生态发展轴线。在城市周边构建布局合理、功能互补、快速联通的卫星镇网络，切实增强城市辐射带动功能。

强化中心城区发展能级。稳步有力推动城市组团建设，南海未来城组团建成南海公园、中华海棠园核心景区，市民中心对外开放；大洋湾生态组团加快功能配套，建设以生态康养为特色的旅游景区；高铁枢纽组团建成综合客运枢纽主站，同步启动片区功能建设；先锋岛生态组团重点打造新洋港景观带，推进沿线片区开发；斗龙港生态组团实施民宿村、实景演艺等生态旅游项目；小马沟组团着力建设生态宜居的城西新空间。持续开展“三治三化”专项行动，市区实施棚户区改造1.7万户、187万平方米，新建安置房1.3万套，整治老旧小区11个，积极推动老居民楼加装电梯。全面实施第Ⅲ防洪区水环境治理，完成33条黑臭河道整治任务，提高城镇污水集中处理水平。逐步削减存量违法建设，实现违法建设“零新增”。提升垃圾分类处理覆盖率，静脉产业园投入使用，市域生活垃圾在全省率先实现全量焚烧发电。加大全国文明城市创建力度，落实“十没有”“二十有”标准，以“绣花”功夫管好城市，提升精细化管理水平。

扎实推进乡村振兴。大力发展现代农业，调整优化农业产业结构和产品结构，鼓励有条件地区推行轮作休耕，新增高标准农田40万亩、设施农业20万亩、综合种养面积30万亩，新建50个万亩以上优质农产品基地，培育优质粮油、规模畜禽、绿色蔬菜等9个百亿元级优势特色产业。推进“互联网+现代农业”，新增投资500万元以上农业龙头企业100家，农产品网络销售突破150亿元。强化农业科技创新与推广，设立农业科技创新基金，每个县（市、区）创办一个省级农业科技园区。继续深化农村改革，稳妥推进农村承包地和宅基地“三权分置”，全面推开农村集体产权制度改革，促进新兴经营主体多元融合发展。全面推进农民群众住房条件改善工作，完成“十镇百村”首批示范镇村建设，改善3万户农民群众住房条件。打造特色田园乡村，高标准建设“四好农村路”，创建20个省、市级特色田园乡村和260个美丽宜居村庄。推进新时代文明实践中心建设，开展文明家庭、文明镇村创建活动，提高乡村文明程度。

加强基础设施建设。构建现代化交通体系，盐徐高铁建成通车，盐通高铁完成线下工程，加快盐泰锡常宜铁路和大丰港、滨海港等铁路支线规划建设，开工建设盐城至射阳高速公路，启动东环路南延至盐淮高速等工程，完善通用机场服务设施。加快市区路桥建设，完成市区内环高架三期、盐渎路跨通榆河大桥、东进路东延、五台山路北延等工程。启动城西潘黄地块土地整理工作。提高市区公交出行通达率，镇村公交线路开通率达90%，更新200辆新能源公交车，加强停车设施建设和智能化管理。推进海绵城市和地下综合管网、管廊建设，实施水利重点工程，增强城市防洪排涝能力。强化能源、民防、信息化等工程建设，推动城乡道路交通、供水供电、供热供气、通讯网络、物流配送等基础设施互联互通、共建共享。

三、深入践行绿色发展理念，增创生态环境新优势

打造绿色生态品牌。大力推进绿色转型、绿色跨越，积极创建国家生态文明建设示范区。全力做好黄海湿地申报世界自然遗产工作，争取申遗一次成功。围绕争创国际湿地城市，以两个自然保护区和一批湿地公园为主体，实施湿地生态修复工程，试点推动生态清淤、聚泥成岛。以创建国家森林城市为抓手，持续推进绿化造林，新增成片林13.1万亩，改造既有林16万亩，建成森林小镇10个、森林村庄100个，城镇绿化覆盖率38.2%，提前一年建成百万亩生态防护林。

提升资源利用效率。严守生态保护红线、环境质量底线、资源利用上线和环境准入负面清单，严守永久基本农田和城市开发边界，深入实施土地综合整治工程。深化生态环境损害赔偿、排污权有偿交易、企业排污许可等制度改革。开展工业企业资源集约利用综合评价，支持各类园区创建绿色园区，新创国家级绿色工厂3家。持续推进“僵尸企业”、闲置土地、低效厂房专项清理行动，盘活资产资源，激活发展动力。制定重点行业、产品和设备能效指导目录，实施一批绿色节能改造项目，实现能源消费总量和强度“双控”目标。

发展湿地生态旅游。积极招引战略性投资主体，发展旅游全产业链项目，建设西部湖荡湿地、东部黄海湿地等一批精品线路、特色景区，打造以“大纵湖—九龙口—马家荡”为代表的旅游景观带，扩大面向高速、高铁沿线特别是上海的旅游市场，争创长三角生态休闲旅游目的地和国家全域旅游示范区。大力培育千鹤湾、大纵湖等10个旅游风情小镇，建设龙冈桃花园等100个精品民宿，荷兰花海争创国家5A级旅游景区。加快建湖九龙口、射阳日月岛、东台黄海森林等项目建设，积极筹办世界湿地生态旅游大会，举办旅游系列宣传活动，开发特色文化旅游产品，促进文化与旅游深度融合。

四、深入践行开放发展理念，开拓对外合作新空间

更大力度开放沿海。全面落实现代海洋经济功能区规划，探索盐场、滩涂、岸线等资源保护与利用新模式，建设国家海洋经济发展示范区。优化沿海产业布局，滨海、响水高起点建设东部沿海现代化大工业基地，大丰、东台、射阳重点推动新能源、电子信息等产业集群发展，建湖、阜宁大力发展生态产业、绿色经济，形成陆海统筹、优势互补的发展格局。发挥淮河生态经济带出海门户作用，加快滨海港工业园区集疏运体系建设，统筹推进宝武、金光、中海油、国家电投等重大项目，打造特大项目集中区资源综合利用循环经济示范基地。推动盐城港扩容提升，加快响水港区、射阳港区两个5万吨级航道建设，开展滨海港区30万吨级深水航道、滨海港直连工程、大丰港区15万吨级深水航道前期工作。加快港城建设，提升配套服务功能。加强海洋渔业资源保护，探索海洋养殖新模式，推进弶港、黄沙港、斗龙港等渔港经济区建设。

更深层次接轨上海。深化产业接轨、市场接轨、科创接轨，学习上海先进理念和管理经验，更好融入长三角高质量一体化发展。推动“一区三基地”建设，加快建设沪苏大丰产业联动集聚区，全面承接孵化上海高新技术产业项目，引进上海亿元以上项目50个。拓展市场空间，加强产销对接，加快上海优质农产品供应基地建设。大力推进盐沪养老一体化，加快上海生态旅游康养基地建设。强化科技合作，用好盐城（上海）国际科创中心，组织园区、企业对接上海高校和科研院所，新签约和实施产学研合作项目200个，加快上海科创成果转化基地建设。广泛开展盐沪社会事业合作，促进优质公共服务资源共建共享。

更高水平做强园区。扎实推进中韩（盐城）产业园建设，全市域、全领域、全方位推动中韩合作。围绕经贸、文化等重点领域，争取落地一批重大项目，核心区外贸出口额、实际利用外资占全市比重分别达到15%、25%，为全省“一带一路”交汇点建设提供有力支撑。借鉴自贸区试点经验，推动开发区与大丰港联动发展，提升对韩合作服务水平，实现集成创新。深化园区“等级创建”，引导重点园区聚焦主导产业，做优基础设施和研发平台，打造一批百亿元级特色产业集群。推动开发区、高新区当好开放型经济龙头，积极推进园区托管改革，乡镇工业园区以创业就业和富民增收为重点发展特色产业。强化园区经济密度和投资强度管控，促进园区高质量发展。

更加主动开放市场。全面提升对外开放水平，注册外资实际到账10亿美元，外贸进出口总额增长5%。支持企业加快“走出去”，推动汽车、风电、光伏等产业海外发展。进一步向民企开放市场，拓展民间投资领域，真心实意支持民营经济发展，制定扶持激励措施，降低税费负担和生产要素成本，充分激发各类市场主体活力，培育更多优秀的本土企业和本地企业家。扎实开展营商环境综合改革，优化亲商富商环境。深化“放管服”改革，推广“一窗通办、一网通行”利企便民不见面服务，80%以上事项“一窗”分类受理，100个高频事项“最多跑一次”。整顿和规范市场秩序，严肃查处各类损害发展环境行为，依法保护企业自主经营权。加快国有企业专业化、市场化改革，优化资产布局，提升经营效益。探索国有企业混合所有制改革，完成经营性事业单位改革改制工作。

五、深入践行共享发展理念，促进民生福祉新提升

完善就业创业和社会保障体系。健全稳就业、促创业、防失业的综合政策体系，吸引盐城籍在外人员回乡创业就业，做好高校毕业生、农村劳动力等重点群体就业服务，鼓励各类经营主体为适能劳动者提供更多就业岗位，鼓励园区和企业更多使用农村转移劳动力，新增城镇就业9万人，城镇登记失业率控制在3%以内。实施“创业盐城”行动计划，拓展“互联网+”等创业新空间，发挥创业富民效应。强化政策富民，健全与经济发展相适应的收入增长机制，提高城乡居民收入。深入实施全民参保计划，做好社保费征收改革，完善社保基金监督体系和风险防控机制，确保社保基金安全平稳运行。大市区实行基本医疗保险统筹，提供优质均衡医疗保障服务。深化国家“救急难”社会救助综合试点，兜住民生底线。

提高公共服务供给质量。坚持尽力而为、量力而行，继续实施20件民生实事，不断增强人民群众的获得感和幸福感。全面落实省基层基本公共服务功能配置标准，确保按期达标。优先发展教育事业，推进教育现代化，扩大优质教育资源供给，加快学前教育优质普惠发展，全市新建、改扩建中小学和幼儿园74所。加强校长、教师队伍建设，培育引进更多名校名师。支持高等院校与地方经济联动发展，提升职业教育水平。深化教育体制改革，推进主城区高中集中统一管理，继续实施普通高中资源增扩。实施智慧教育工程，推广中小学校“弹性离校、课后服务、集中就餐”，推动校园体育设施有序向社会开放。深入推进“健康盐城”建设，深化医疗卫生体制改革，加强医疗资源供给。大力提升县级医疗机构服务能力，县域就诊率达90%以上。实施市中医院扩建、市一院南区二期、市儿童医院等重点项目，新增省示范镇卫生院6个、示范村卫生室60个，启动全民健康体检，加强药品、疫苗监管，提高公共医疗服务水平。创建国家全民运动健身模范市，举办市第八届运动会等赛事活动。鼓励社会资本进入养老服务领域，提供居家和社区养老、机构养老、医养结合等多样化服务。加大残疾人关爱力度，落实保障政策和救助制度。加强农贸市场建设，继续推进“厕所革命”，完善社区服务设施功能。

推动文化繁荣发展。积极培育和践行社会主义核心价值观，大力宣传“中国好人”、生态卫士等先进典型，提升公民文明素质和社会文明程度。建强文化阵地，完成新四军纪念馆展陈提升改造工程，完善村（社区）综合性文化服务中心，新建60家阅读新空间，建设一批24小时自助图书馆（柜）。积极发展文化产业，规划建设文化产业集聚区，扶持发展一批文化企业。鼓励文艺创作，传承红色文化，支持淮剧、杂技等文化团体发展，打造一批特色文化街区，丰富群众精神文化生活。

继续加强和创新社会治理。创新升级社会治安防控体系，推进扫黑除恶专项斗争向纵深发展，深化“黄海行动”“梳网清格行动”，进一步增强人民群众安全感。健全社会矛盾多元化调解机制，依法分类处理信访问题。加强应急管理体系建设，增强气象等灾害监测和预警能力，有效应对和处置各类突发事件。深入推进“七五”普法，提升全民法律素养。发挥社会组织作用，推动社会治理重心下移和网格化管理，提升社区治理服务能力，实现政府治理与社会调节、居民自治良性互动。扎实抓好双拥共建，做好退役军人保障工作，营造安定有序的发展环境。

各位代表，全面建成小康社会，实现第一个百年奋斗目标，是我们党向人民、向历史作出的庄严承诺。我们必须强化责任担当，坚持底线思维，打好打赢“三大攻坚战”，使全面建成小康社会得到人民认可、经得起历史检验。

坚决打好防范化解重大风险攻坚战。采取更加有力有效措施，严控金融、社会等领域的风险隐患。加强政府投资项目源头管理，加大政府债务化解力度，确保不发生政府性债务风险，确保不新发生违规融资行为，确保债务规模和债务率得到有效控制。采取园区托管、债务置换等方式，有效化解镇村债务。深化地方金融监管体制改革，严厉打击非法金融活动，推动农民资金互助合作社规范管理。强化社会面防控，全面排查整治重点领域、重点行业、重点群体风险隐患。严格落实安全生产责任制，遏制较大事故，杜绝重特大事故，确保人民群众生命财产安全。

坚决打好精准脱贫攻坚战。聚焦低收入人口、经济薄弱村和重点片区，深入推进精准脱贫，因地制宜发展特色高效产业，壮大村级集体经济，拓宽农民增收渠道，完成建档立卡低收入人口省定脱贫任务，滨海、响水两县和省定经济薄弱村全部实现脱贫，达到“摘帽”标准。严格对照“两不愁、三保障”要求，强化兜底保障，全面落实教育、医疗、低保等扶贫政策，促进政策性保障与扶贫有效衔接。推进“阳光扶贫”，严格扶贫资金监管，提高资金使用效率。继续做好援疆、援青以及与陕西铜川、辽宁阜新对口协作工作。

坚决打好污染防治攻坚战。坚持末端治理与源头防控“两手抓”，以中央和省环保督察交办问题整改为重点，开展“263”“绿盾”“散乱污企业”整治等专项行动，打好蓝天、碧水、净土保卫战。加强工业、能源等领域污染治理，持续提升空气质量，PM2.5年均浓度小于41微克/立方米。大力推进水环境整治，推动河（湖）长制常态化，开展黄海入海水系全流域治理，加快畜禽水产养殖、近海水域等污染防治，入海河流水质提升、稳定达标。实施土壤污染治理与修复工程，促进土壤可持续利用。持续实施化工园区专项整治、化工企业“四个一批”专项行动，坚决关停环保隐患突出、无法通过整治验收的化工生产企业。

建设人民满意的政府

新的形势和任务，对政府自身建设提出新的更高要求。我们将始终坚持用习近平新时代中国特色社会主义思想武装头脑、指导实践、推动工作，以人民为中心，不忘初心、牢记使命，切实提高政治站位，树牢“四个意识”，坚定“四个自信”，坚决做到“两个维护”，更好发挥政府作用，更加让人民满意，努力当好新时代的答卷人。

深入解放思想，打造创新政府。始终保持开拓创新的勇气、敢为人先的锐气和蓬勃向上的朝气，紧盯走在前列的目标定位，在坚决守住稳定、安全、廉政、生态四条底线的前提下，创造性地开展工作。注重学习新知识、新政策、新经验，着力增强“八个本领”，更多采取改革的办法和市场化、法治化手段，破解发展难题，推动经济社会高质量发展。

勇于担当作为，打造效能政府。坚持在市委领导下开展工作，按照中央和省市决策部署，坚决有力抓好贯彻落实，全面提升政府工作执行力。围绕推动经济发展、改革开放、城乡建设、文化建设、生态环境、人民生活“六个高质量”发展要求，实行清单式管理，确保有部署、有落实、有督查、有结果。大力弘扬求真务实作风，着力加强效能建设，雷厉风行、紧张快干，做细做实做好各项工作。强化督查问责和绩效考评，落实“三项机制”要求，营造敢负责、勇担当、善成事的浓厚氛围。

加快职能转变，打造服务政府。稳妥有序推进政府机构改革，优化机构设置和职能配置，为社会提供更多更优的公共服务。坚持以信息化提升工作效能，加快政务云门户建设，推动“互联网+政务服务”“互联网+为民服务”。完善网上服务与实体大厅相结合的一体化政务服务模式，实现高频服务事项移动端办理，精心打造城市智能门户，努力让群众享受到方便快捷的公共服务。以更实举措优化发展环境，深化“暖企行动”，构建“亲”“清”新型政商关系，让各类市场主体放心创业、安心发展。

依法履行职责，打造法治政府。坚持依宪施政、依法行政，突出抓好集体学法、出庭应诉等制度落实，增强政府及工作人员学法用法能力。修订完善政府工作规则，健全政府决策机制，强化重大行政决策合法性审查，推进依法、科学、民主决策。深化综合行政执法改革，建立健全权责统一、权威高效的体制机制。主动接受人大法律监督和工作监督、政协民主监督，提高建议和提案的办成率、满意度，更加重视司法监督、审计监督、舆论监督。推进重点领域政务公开，做好政策解读回应，提高政府工作公信力。

持续改进作风，打造勤廉政府。坚决贯彻执行中央八项规定及实施细则，以永远在路上的执着，严格落实全面从严治党各项要求。紧盯突出问题，持之以恒深化“四风”整治，坚决破除形式主义、官僚主义。大力弘扬爱岗敬业、只争朝夕的干事创业精神，和全市人民一起拼搏、一起奋斗，实干在前。全面落实党风廉政建设责任制，强化部门单位的主体责任，健全内控机制，防控廉政风险，严格控制“三公”经费和一般性支出。坚决有力整改中央、省委巡视以及市委巡察反馈问题，主动接受纪委、监委监督，配合做好“正风肃纪看机关”等专项行动，以为民务实、廉洁高效的良好形象取信于民。

各位代表！时代因奋斗而常新，城市因梦想而远行。让我们更加紧密地团结在以习近平同志为核心的党中央周围，在省委省政府和市委的坚强领导下，进一步解放思想、开拓创新，锐意进取、担当实干，以优异成绩庆祝中华人民共和国成立70周年，为高水平全面建成小康社会、高质量发展走在苏北苏中前列而不懈奋斗！
